# Hyperscaling Violation in Ising Spin Glasses

**DOI:** 10.3390/e21100978

**Published:** 2019-10-08

**Authors:** Ian A. Campbell, Per H. Lundow

**Affiliations:** 1 Laboratoire Charles Coulomb (L2C), UMR 5221 CNRS-Université de Montpellier, 34095 Montpellier, France; ian.campbell@umontpellier.fr; 2 Department of Mathematics and Mathematical Statistics, Umeå University, SE-901 87 Umeå, Sweden

**Keywords:** spin glasses, random interactions, scaling, hyperscaling, 75.50.Lk, 75.40.Mg, 05.50.+q

## Abstract

In addition to the standard scaling rules relating critical exponents at second order transitions, hyperscaling rules involve the dimension of the model. It is well known that in canonical Ising models hyperscaling rules are modified above the upper critical dimension. It was shown by M. Schwartz in 1991 that hyperscaling can also break down in Ising systems with quenched random interactions; Random Field Ising models, which are in this class, have been intensively studied. Here, numerical Ising Spin Glass data relating the scaling of the normalized Binder cumulant to that of the reduced correlation length are presented for dimensions 3, 4, 5, and 7. Hyperscaling is clearly violated in dimensions 3 and 4, as well as above the upper critical dimension D=6. Estimates are obtained for the “violation of hyperscaling exponent” values in the various models.

## 1. Introduction

It has been tacitly or explicitly assumed that Edwards–Anderson Ising Spin Glasses (ISGs), where the quenched interactions are random, follow the same basic scaling and Universality rules as in the canonical Ising models, whose properties are understood in great detail through Renormalization Group Theory (RGT). Here we will present numerical evidence for hyperscaling violation in ISGs. A textbook definition of hyperscaling is: “Identities obtained from the generalised homogeneity assumption involving the space dimension *D* are known as hyperscaling relations.” [1]. The hyperscaling relations valid in canonical Ising models below the upper critical dimension are: 2−α=Dν, and 2Δ=Dν+γ, where Δ=γ+β is the “gap” exponent associated with the critical behavior of the higher field derivatives of the free energy [2,3]. The two hyperscaling relations are linked through the Essam–Fisher relation α+Dν−2Δ=2.

Hyperscaling “collapses” in Ising models in dimensions above the upper critical dimension D=4, where the critical exponents take their mean-field values: γ=1, ν=1/2, α=0, and Δ=3/2. Hyperscaling was predicted by Schwartz to break down also in quenched systems with random interactions [4]. The breakdown of hyperscaling in the 3D Random Field Ising model (RFIM) has been extensively studied [5,6,7,8]. The first hyperscaling relation in this model is re-written 2−α=(D−θ)ν where θ is the “violation of hyperscaling exponent” [5] with θ∼1.47 in the 3D RFIM [9,10]. Logically, the second hyperscaling relation should simultaneously become 2Δ=(D−θ)ν+γ.

Though not conventionally written this way, in the standard Ising models above D=4 equivalent modified hyperscaling relations 2−α=(D−θ)ν=2 and 2Δ=(D−θ)ν+γ=3 can be seen by inspection to be consistent with the mean field exponents plus a violation exponent θ=D−4.

Ising spin glasses (ISGs) are also systems with quenched randomness in which hyperscaling might be expected to break down, from a generalization of Schwartz’s argument. The exponent α in ISGs is always strongly negative and so is very hard to estimate directly; we will be concerned only with the second hyperscaling relation. We are not aware of any tests of hyperscaling in ISGs.

## 2. Scaling

As usual the Hamiltonian is $H=-\sum_{ij}J_{ij}S_{i}S_{j}$. The sum is taken over all nearest-neighbour spins and the $J_{ij}$ are the interaction strengths. For Ising models we let $J_{ij}=1$ but for ISGs the $J_{ij}$ are drawn from a symmetric distribution ($\langle J_{ij}\rangle =0$) normalized to $\langle J_{ij}^{2}\rangle =1$. Also, while the inverse temperature is β=1/T for the Ising case one should keep in mind that for ISGs it is $\beta ={\left(\langle J_{ij}^{2}\rangle /T^{2}\right)}^{1/2}$, which is consistent with the Ising case.

The magnetisation $m=L^{-d}\sum_{i}S_{i}$ has the role of order parameter for Ising models but for ISGs we use the spin overlap parameter

(1) $$q=\frac{1}{L^{d}}\sum_{i}S_{i}^{A}S_{i}^{B}$$

where *A* and *B* indicate two spin systems on the same interaction realizations (samples). The susceptibility is then $\chi =L^{d}\langle m^{2}\rangle$ for Ising models while for ISGs we use $\chi =L^{d}\left[\langle q^{2}\rangle \right]$, where [⋯] denotes the sample average.

The Binder cumulant [11], a normalized form of the kurtosis, is defined as

(2) $$g=\frac{1}{2}\left(3-\frac{\langle m^{4}\rangle }{{\langle m^{2}\rangle }^{2}}\right)$$

for Ising models and as

(3) $$g=\frac{1}{2}\left(3-\frac{\left[\langle q^{4}\rangle \right]}{{\left[\langle q^{2}\rangle \right]}^{2}}\right)$$

for spin glasses. Finally, the standard finite-*L* definition for the second-moment correlation length ξ(β,L) through the Fourier transformation of the correlation function is used, see Ref. [12] Equation (8),

(4) $$\xi =\frac{\sqrt{\chi /F-1}}{2\sin(\pi /L)}$$

and

(5) $$F_{k}=\frac{1}{L^{d}}\langle {\left(\sum_{x}q_{x}\;\exp\left(2\pi ix_{k}/L\right)\right)}^{2}\rangle$$

where $q_{x}=S_{x}^{A}S_{x}^{B}$ for ISGs and just $S_{x}$ for Ising models. Here, $x_{k}$ is the *k*th spin coordinate so we let $F=(F_{1}+\cdots +F_{D})/D$ be the average over the *D* directions. For ISGs we then also take the sample average of *F* in Equation (4). The ISG data were gathered using exchange Monte Carlo [13] and the Ising data with the Wolff-cluster technique.

The conventional RGT-based approach to analyses of numerical simulation data is to use the reduced temperature $t=\left(T-T_{c}\right)/T_{c}$ as the thermal scaling variable, together with the principal observables χ(t,L), ξ(t,L) and g(t,L). This approach is then tailored to the critical region. However, at high temperatures the scaling variable *t* diverges and ξ(t,L) tends to zero, so analyses of the entire paramagnetic regime is not possible without introducing diverging correction terms. In addition, in symmetric-interaction ISGs the relevant interaction-strength parameter is $\langle J_{ij}^{2}\rangle$ so the ISG thermal scaling variable should depend on the square of the temperature; this basic point was made some thirty years ago [14,15] but has been ignored since in most ISG simulation data analyses.

A rational scaling approach which takes in the entire paramagnetic region, thus including both the finite-size scaling regime (FSS, L≪ξ(β,∞)) and the thermodynamic limit regime (ThL, L≫ξ(β,∞)), can be based on the Wegner scaling expression for the bulk Ising susceptibility [16]

(6) $$\chi \left(\tau \right)=C_{\chi }\tau^{-\gamma }\left(1+a_{\chi }\tau^{\nu \omega }+b_{\chi }\tau +\cdots \right)$$

where $\tau =1-\beta /\beta_{c}$ in Ising models with β the inverse temperature [17]. (The Wegner expression is often mis-quoted with *t* replacing τ). The terms inside (⋯) are scaling corrections, where νω is the leading thermal correction exponent, which is identical for all observables within a universality class.

At infinite temperature τ=1, and for all spin-1/2 models, χ(τ) tends to 1; hence, for the susceptibility the whole paramagnetic region can generally be covered to good precision when a few mild Wegner correction terms are included. (To obtain infinite precision an infinite number of correction terms are of course needed, just as in standard FSS analyses where perfect precision in principle requires an infinite number of size dependent correction terms [18]).

For ISG models with symmetric interaction distributions an appropriate thermal scaling variable is $\tau =1-{\left(\beta /\beta_{c}\right)}^{2}$, which then can be used in the Wegner expression [14,15,19,20]. In the ThL regime L≫ξ(β) the properties of a finite-size sample, if normalized correctly, are independent of *L* and so are the same as those of the infinite-*L* model. A standard rule of thumb for the approximate onset of the ThL regime is L≳7ξ(β,L) and this is easily identified in the simulation data. A huge benefit with this approach is that the ThL numerical data can be readily dovetailed into High Temperature Series Expansion (HTSE) values obtained from exact series terms (in practice limited to a finite number of terms). This is lost when the conventional FSS thermal scaling variable *t* is used.

To apply the Wegner formalism to other observables *Q* than χ in spin-1/2 models, it is convenient to impose the rule that each observable should be normalized in such a way that that the infinite temperature limit is $Q_{n}\left(\tau =1\right)\equiv 1$, without the critical limit exponent being modified. For the spin-1/2 [reduced] susceptibility no normalization is required as this condition is automatically fulfilled, with a temperature dependent effective exponent γ(τ)=∂ln[χ(τ,L)]/∂lnτ both in Ising models, and in ISGs with the appropriate τ.

The reduced second-moment correlation length is defined as $\xi \left(\tau ,L\right)/\beta^{1/2}$ in Ising models and as ξ(τ,L)/β in ISG models [20]. Note that the critical-limit ThL exponent ν is unaltered by this normalization (models with zero-temperature critical points are a special case [21]). From exact and general HTSE for either model this reduced correlation length tends to 1 at infinite temperature [2,19].

The temperature-dependent effective exponent is then defined as $\nu \left(\tau \right)=\partial \ln[\xi \left(\tau ,L\right)/\beta^{1/2}]/\partial \ln\tau$ in Ising models and ν(τ)=∂ln[ξ(τ,L)/β]/∂lnτ in ISG models. A Wegner-like relation again applies with appropriate correction terms. With this definition the temperature-dependent effective ν(τ) usually turns out to remain close to the critical value, except at high temperatures where it may be significantly modified by the corrections.

The temperature-dependent effective exponents γ(τ) and ν(τ) are well-behaved over the entire paramagnetic regime, with exact infinite-temperature hypercubic-lattice limits $\gamma \left(1\right)=2D\beta_{c}$ and $\nu \left(1\right)=D\beta_{c}$ for Ising models. For the ISG models they are $\gamma \left(1\right)=2D\beta_{c}^{2}$ and $\nu \left(1\right)=\left(D-K/3\right)\beta_{c}^{2}$, where *K* is the kurtosis of the interaction distribution.

## 3. Hyperscaling

The second field derivative of the bulk susceptibility $\chi_{4}\left(\beta \right)$ (fourth derivative of logZ, also called the non-linear susceptibility) in a hypercubic lattice is directly related to the ThL Binder cumulant for finite *L* through

(7) $$2g\left(\beta ,L\right)=\frac{-\chi_{4}}{L^{D}\chi^{2}}=\frac{3{\langle m^{2}\rangle }^{2}-\langle m^{4}\rangle }{{\langle m^{2}\rangle }^{2}}$$

see Ref. [22] Equation (10.2). It can be noted that in any spin-1/2 Ising system the infinite-temperature limit (i.e., independent spins) for the Binder cumulant is g(0,N)≡1/N, where N is the number of spins; as $N=L^{D}$ for a hypercubic lattice, at infinite temperature $L^{D}g\left(\tau ,L\right)\equiv 1$. Thus this normalized Binder cumulant also obeys the high-temperature limit rule for normalized observables introduced above.

For Ising models in the ThL (bulk or L≫ξ(τ)) regime, assuming hyperscaling the critical exponent for the second field derivative of the susceptibility $\chi_{4}\left(\beta \right)$ is [2]

(8) $$\gamma_{4}=\gamma +2\Delta =D\nu +2\gamma$$

Thus the bulk $\chi_{4}\left(\beta \right)/\left(2\chi {\left(\beta \right)}^{2}\right)$ or the ThL normalized Binder cumulant $L^{D}g\left(\beta ,L\right)$ scales with the critical exponent (νD+2γ)−2γ=νD, together with appropriate Wegner correction terms.

The standard “dimensionless renormalized coupling constant” can be defined as

(9) $$G_{4}\left(\beta \right)=\frac{\chi_{4}\left(\beta \right)}{\xi {\left(\beta \right)}^{D}\chi {\left(\beta \right)}^{2}}=\frac{L^{D}g\left(\beta ,L\right)}{2\xi {\left(\beta ,L\right)}^{D}}$$

(other normalizations are also used [2]). It should be noted that even in the case of the canonical 2D Ising model [23] the infinite-*L* value of $G_{4}\left(\beta_{c}\right)$ at criticality depends strongly on the order in which the limits are taken: the ThL limit $\left(L\to \infty ,\beta \to \beta_{c}\right)$ or the FSS limit $\left(\beta \to \beta_{c},L\to \infty \right)$. Also, in order for the infinite-*L* Ising $G_{4}$ to become regular analytic to β=0 the normalized Ising form ${\left(\beta_{c}/\beta \right)}^{D/2}G_{4}\left(\beta \right)$ should be used, see Ref. [2] Equation (42). This modification is strictly equivalent to replacing in Equation (9) ξ(β,L) by $\xi \left(\beta ,L\right)/\beta^{1/2}$ which is the reduced correlation length introduced above.

**Figure 1:**
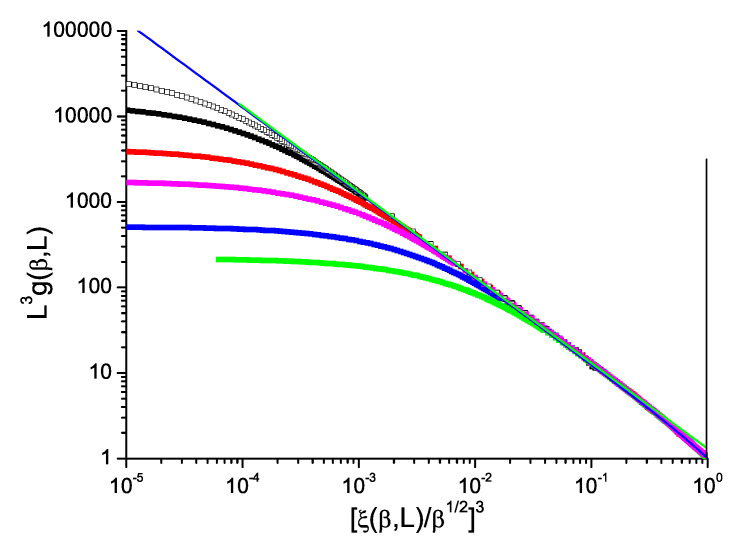
(Color on line) Dimension 3 simple cubic Ising model. Normalized Binder cumulant $L^{3}g\left(\beta ,L\right)$ against reduced correlation length to the power 3, $1/{\left(\xi \left(\beta ,L\right)/\beta^{1/2}\right)}^{3}$. Sample sizes: L=32, 24, 16, 8, 6 (top to bottom). Blue straight line: slope −1.00. In this and all following figures each line for fixed *L* begins to bend over towards horizontal when it leaves the ThL regime L>>ξ(β).

From the hyperscaling rule, the critical exponents for $L^{D}g\left(\tau ,L\right)$ and ${\left(\xi \left(\tau ,L\right)/\beta^{1/2}\right)}^{D}$ are both Dν. Figure 1 shows a plot of $L^{3}g\left(\tau ,L\right)$ against ${\left(\xi \left(\tau ,L\right)/\beta^{1/2}\right)}^{-3}$ for the 3D simple cubic spin-1/2 Ising model (see Ref. [24] for details of the simulations). It can be seen that within the statistics the ThL data are consistent with a limiting critical slope ≡−1 and an intercept $G_{4}\beta_{c}^{3/2}/2∼1.23$, with corrections coming into play at high temperatures, in full agreement with the hyperscaling rule. It should be noted that in this form of plot neither the critical inverse temperature value $\beta_{c}$ nor the critical exponent value ν need to be introduced.

For the 5D Ising model, in an equivalent ThL plot covering the entire paramagnetic temperature regime the breakdown of standard hyperscaling leads to a critical exponent for the normalized Binder cumulant which is not Dν=5/2 but 2 [25], i.e., (D−θ)ν with a hyperscaling violation exponent θ=1 as discussed above.

## 4. Ising Spin Glasses

**Figure 2:**
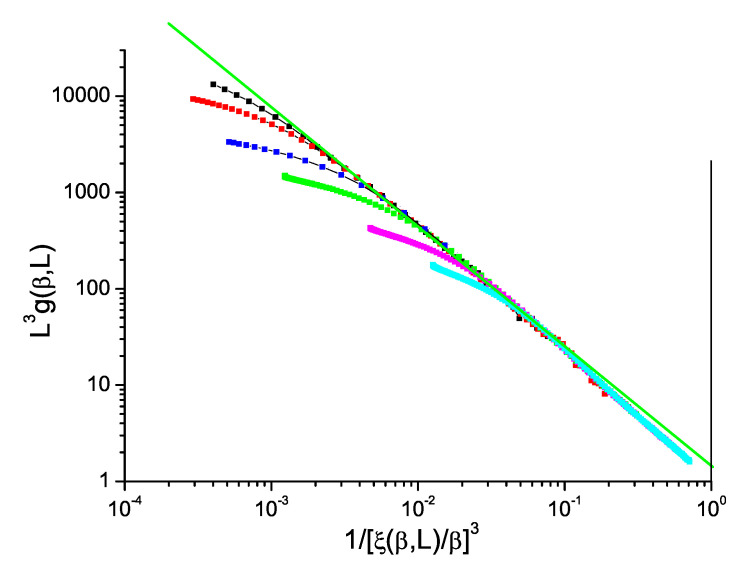
(Color on line) Dimension 3 simple cubic bimodal ISG model. Normalized Binder cumulant $L^{3}g\left(\beta ,L\right)$ against reduced correlation length to the power 3, $1/{\left(\xi \left(\beta ,L\right)/\beta \right)}^{3}$. Sample sizes: L=32, 24, 16, 12, 8, 6 (top to bottom). Green straight line: slope −1.27.

**Figure 3:**
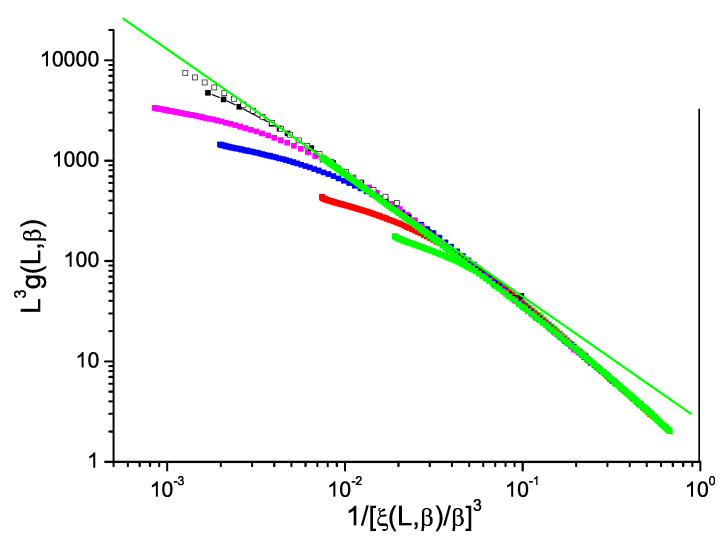
(Color on line) Dimension 3 simple cubic Gaussian ISG model. Normalized Binder cumulant $L^{3}g\left(\beta ,L\right)$ against reduced correlation length to the power 3, $1/{\left(\xi \left(\beta ,L\right)/\beta \right)}^{3}$. Sample sizes: L=32, 24, 16, 12, 8, 6 (top to bottom). Green straight line: slope −1.23.

**Figure 4:**
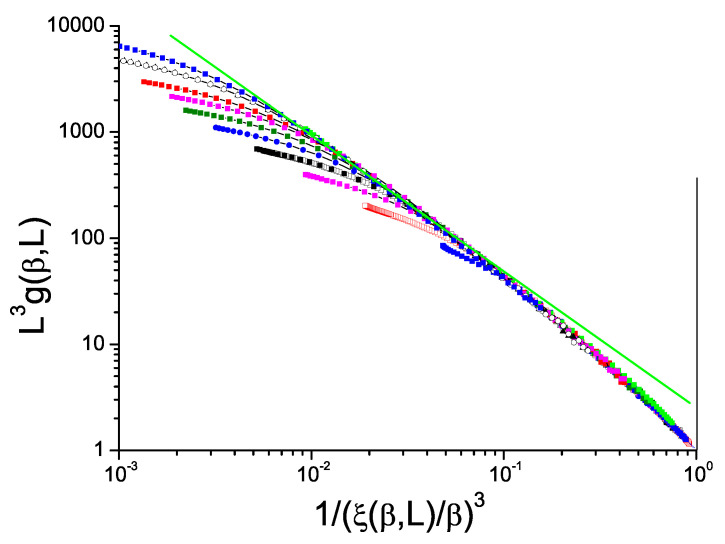
(Color on line) Dimension 3 face-centered cubic Laplacian ISG model. Normalized Binder cumulant $L^{3}g\left(\beta ,L\right)$ against reduced correlation length to the power 3, $1/{\left(\xi \left(\beta ,L\right)/\beta \right)}^{3}$. Sample sizes: L=28, 24, 20, 18, 16, 14, 12, 10, 8, 6 (top to bottom). Green straight line: slope −1.27.

The normalized Binder cumulant against reduced correlation length to the power *D* for ISGs can be displayed in just the same way as for the Ising model data in Figure 1. We show in Figure 2 and Figure 3 data for the standard bimodal and Gaussian interaction ISGs on the 3D simple cubic lattice, and in Figure 4 for a more exotic model, the Laplacian-interaction ISG on a face-centered cubic lattice.

In each case the ThL data can be seen to have a critical limit constant slope with corrections coming into play at high temperatures. The value of the limit slope is in each case distinctly stronger than the standard hyperscaling value −1, demonstrating that ${\left(\xi \left(\beta ,L\right)/\beta \right)}^{3})$ and $L^{3}g\left(\beta ,L\right)$ have different critical exponents; there is a violation of hyperscaling. With the same formalism as above, the value of the slope in each Figure can be taken to be equal to −(D−θ)/D=θ/D−1 with θ∼−0.80, −0.70 and −0.80 respectively for the three 3D ISG models.

**Figure 5:**
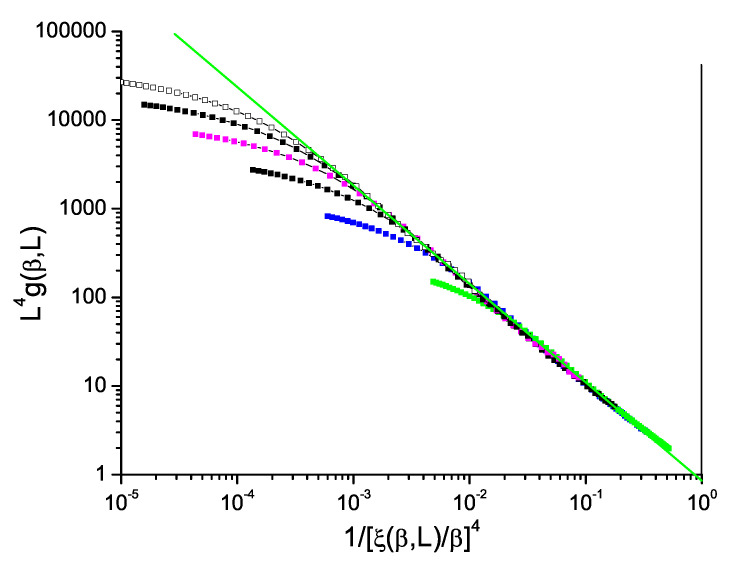
(Color on line) Dimension 4 hypercubic bimodal ISG model. Normalized Binder cumulant $L^{4}g\left(\beta ,L\right)$ against reduced correlation length to the power 4, $1/{\left(\xi \left(\beta ,L\right)/\beta \right)}^{4}$. Sample sizes: L=14, 12, 10, 8, 6, 4 (top to bottom). Green straight line: slope −1.12.

**Figure 6:**
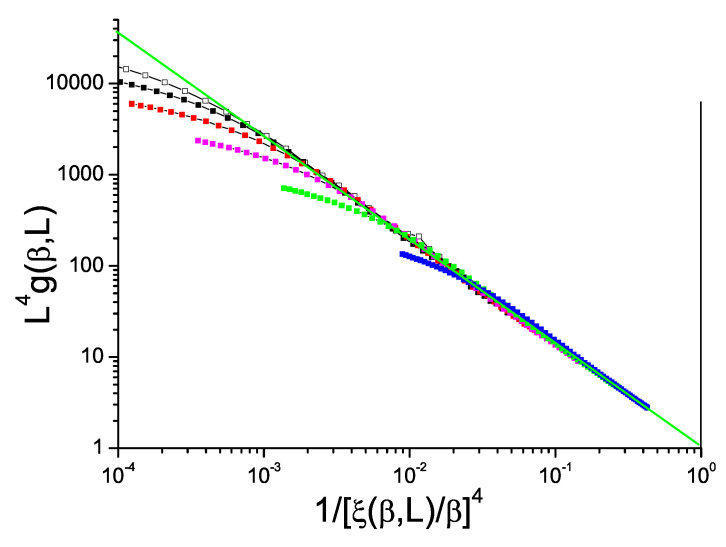
(Color on line) Dimension 4 hypercubic Gaussian ISG model. Normalized Binder cumulant $L^{4}g\left(\beta ,L\right)$ against reduced correlation length to the power 4, $1/{\left(\xi \left(\beta ,L\right)/\beta \right)}^{4}$. Sample sizes: L=14, 12, 10, 8, 6, 4 (top to bottom). Green straight line: slope −1.13.

**Figure 7:**
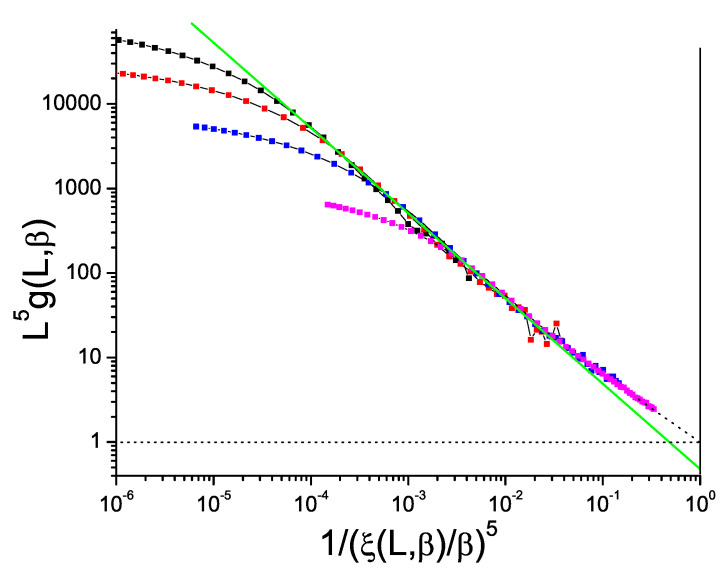
(Color on line) Dimension 5 hypercubic bimodal ISG model. Normalized Binder cumulant $L^{5}g\left(\beta ,L\right)$ against reduced correlation length to the power 5, $1/{\left(\xi \left(\beta ,L\right)/\beta \right)}^{5}$. Sample sizes: L=10, 8, 6, 4 (top to bottom). Green straight line: slope −1.00.

**Figure 8:**
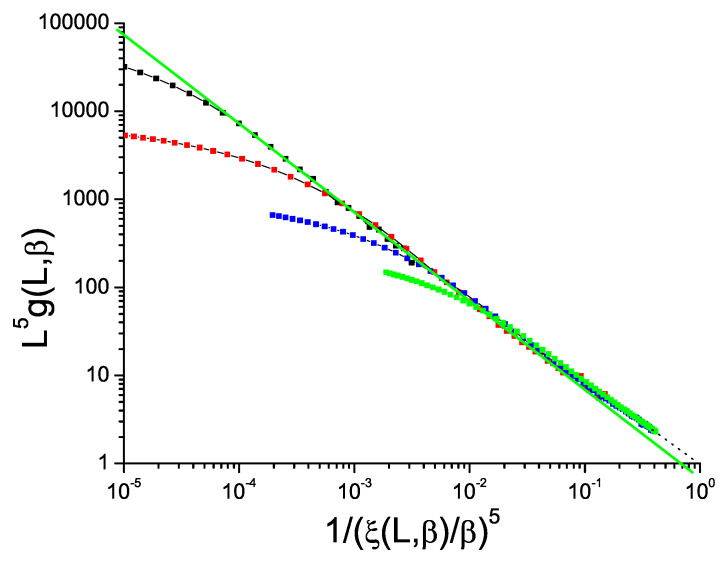
(Color on line) Dimension 5 hypercubic Gaussian ISG model. Normalized Binder cumulant $L^{5}g\left(\beta ,L\right)$ against reduced correlation length to the power 5, $1/{\left(\xi \left(\beta ,L\right)/\beta \right)}^{5}$. Sample sizes: L=10, 8, 6, 4 (top to bottom). Green straight line: slope −1.00.

Data for the bimodal and Gaussian 4D models, Figure 5 and Figure 6, also show hyperscaling violations with violation exponents θ∼−0.48 and −0.52 respectively, rather weaker than in 3D. For the bimodal and Gaussian 5D models, Figure 7 and Figure 8, the limiting slopes are close to −1 and any violation of hyperscaling is too weak to be observed.

**Figure 9:**
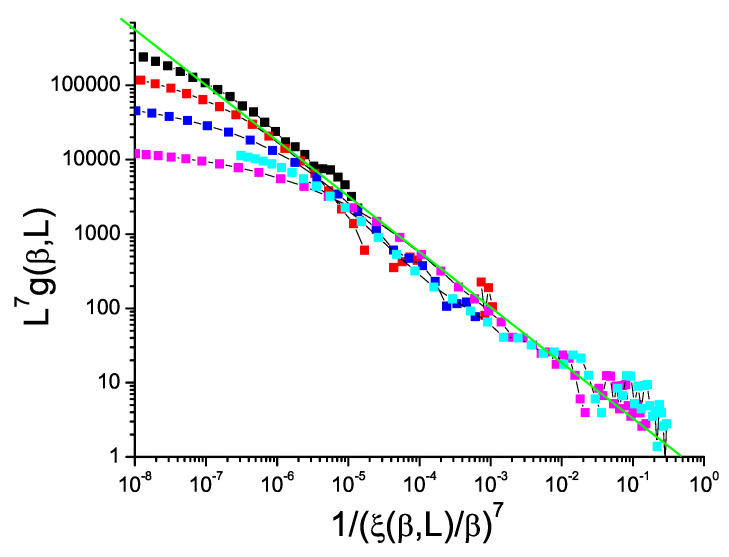
(Color on line) Dimension 7 hypercubic bimodal ISG model. Normalized Binder cumulant $L^{7}g\left(\beta ,L\right)$ against reduced correlation length to the power 7, $1/{\left(\xi \left(\beta ,L\right)/\beta \right)}^{7}$. Sample sizes: L=7, 6, 5, 4, 3 (top to bottom). Green straight line: slope −0.75.

Finally, in the bimodal 7D ISG (above the ISG critical dimension D=6), the equivalent plot, Figure 9, is much more noisy than for the lower dimensions simply because by this dimension the number of spins in each individual sample becomes very large leading to practical limitations, particularly for the Binder cumulant. Nevertheless the slope of the plot can be seen to be lower than −1 so, as for the 5D Ising model, the violation exponent θ∼1.75 is positive.

## 5. Conclusions

For the canonical simple cubic Ising model, from data presented in the form of the normalized Binder cumulant $L^{3}g\left(\tau ,L\right)$ against the reduced correlation length to power *D*, ${\left[\xi /\beta^{1/2}\right]}^{D}$, the observed critical-limit scaling is fully consistent with the expected standard hyperscaling log-log slope of −1 (plus mild corrections at high temperatures). The equivalent data plots for ISG models in various dimensions show violations of hyperscaling with violation exponents which evolve regularly with dimension from strongly negative for D=3 to strongly positive for D=7, passing though zero near the upper critical dimension.
